# Typhus Group Rickettsiosis and the Dengue Diagnostic Paradox: Low Laboratory-Confirmed Dengue Amid the Americas’ Largest Epidemic

**DOI:** 10.3390/idr18040074

**Published:** 2026-07-14

**Authors:** Manuel Sierra, Elsa Palou, Fernando Baires, Helen Hoffman, Heike Hesse, Miguel Sierra-Hoffman, Amy C. Madril

**Affiliations:** 1 Faculty of Medical Sciences, Central American Technological University (UNITEC), Tegucigalpa 11101, Honduras; mass_honduras_2006@yahoo.com; 2 Department of Internal Medicine and Infectious Disease, Honduran Medical Center, Tegucigalpa 11101, Honduras; elsapalou27@hotmail.com; 3 Faculty of Medical Sciences, Autonomous University of Honduras, Tegucigalpa 11101, Honduras; ferbaires93@hotmail.com; 4 Faculty of Medical Sciences, Latin University of Costa Rica Powered by Arizona State University, San Jose 11501, Costa Rica; sierrahelen7@gmail.com; 5 One Health Research Institute, Central American Technological University (UNITEC), Tegucigalpa 11101, Honduras; hhesse.neurologia@gmail.com; 6 Department of Infectious Disease and Internal Medicine, Sam Houston State University, Conroe, TX 77304, USA; 7 Department of Hospital Medicine, El Campo Memorial Hospital, El Campo, TX 77437, USA

**Keywords:** typhus, dengue, rickettsia, rickettsia typhus group, Central America, South Texas

## Abstract

Background: Rickettsial diseases, particularly typhus group rickettsioses, remain underrecognized across the Americas despite ecological conditions favorable for transmission. Concurrently, Central America is experiencing one of its largest dengue epidemics, with an unexpectedly low proportion of laboratory-confirmed cases. Objective: We aimed to explore whether typhus group rickettsioses may contribute to dengue-like febrile illness in Central America in the context of low dengue diagnostic confirmation. Materials and Methods: An analytical narrative review was conducted integrating secondary epidemiological data from the Pan American Health Organization (PAHO) Arbovirus Surveillance Portal and the peer-reviewed literature. Data from seven Central American countries (2024) were analyzed to estimate the proportion of laboratory-confirmed dengue cases. A focused literature review examined epidemiological, clinical, and ecological evidence supporting rickettsial transmission. Results: Of 543,006 reported dengue cases in Central America in 2024, only 62,285 (11%) were laboratory-confirmed. Evidence from Costa Rica and regional serologic studies suggests ongoing rickettsial transmission. Ecological and socioeconomic conditions—including vector abundance, peridomestic reservoirs, and climate variability—mirror those of South Texas, where murine typhus is endemic. Conclusions: The discrepancy between reported and confirmed dengue cases suggests a potential diagnostic gap. Typhus group rickettsioses represent a plausible, underrecognized contributor to febrile illness in Central America. Strengthening surveillance, diagnostics, and clinical awareness is essential to address this hidden burden.

## 1. Introduction

Rickettsial diseases remain an underrecognized and neglected component of emerging infectious threats throughout the Americas, despite their historical presence and substantial potential to cause morbidity in tropical and subtropical settings. The comprehensive 18-year review of murine typhus in South Texas conducted by Howard and Fergie [1] offers valuable insights into the epidemiologic patterns, clinical manifestations, and public health implications of *Rickettsia typhi* infections among pediatric populations. Their work highlights a persistent endemic focus embedded within ecological and socioeconomic contexts that closely mirror those of Central America—particularly along the Pacific and Atlantic lowlands of Honduras, El Salvador, Nicaragua, and Costa Rica. These parallels warrant renewed regional attention to diagnostic, surveillance, and vector control deficiencies. When these observations are viewed alongside the strikingly low dengue test positivity rate—only 11%, as shown in Table 1—they prompt a serious question. Are these clinically diagnosed dengue cases truly dengue, or could a clinically similar febrile illness be masquerading as a dengue-like syndrome without laboratory confirmation? An anomaly of this magnitude warrants careful investigation rather than passive acceptance.

Murine typhus, caused by *R. typhi* and transmitted primarily by the cat flea (*Ctenocephalides felis*) and the oriental rat flea (*Xenopsylla cheopis*), represents a classic flea-borne zoonosis whose incidence reflects interactions between urbanization, vector ecology, and socioeconomic vulnerability [3,4]. Historically endemic to coastal areas of the southeastern United States during the early to mid-20th century [5], its reemergence in South Texas [1] and sporadic detection in California [6] underscore the continuity of an eco-epidemiological corridor that extends into Mesoamerica. The recent identification of *R. typhi* in Costa Rica [7] and serologic evidence of exposure to spotted fever group and typhus group rickettsiae in several Central American countries [8,9] suggest ongoing, silent transmission in areas where diagnostic infrastructure is limited and surveillance remains largely passive.

Central America encompasses a range of ecological gradients—from humid tropical lowlands to semi-arid intermountain valleys—that support abundant populations of peridomestic reservoirs and vectors. The combination of urban–rural interface zones, proliferation of stray animals, inadequate solid waste management, and climate-sensitive vector dynamics creates ideal conditions for flea-borne transmission cycles similar to those documented in South Texas [1,5,9]. Temperature, rainfall, and humidity patterns directly influence flea survival and rodent population density, while socioeconomic factors—such as informal housing, domestic animal infestation, and limited access to veterinary care—exacerbate human exposure risks [10]. These interacting determinants sustain an ecological niche highly favorable to *R. typhi* persistence yet remain underappreciated due to limited clinical awareness and public health prioritization.

## 2. Materials and Methods

This study was conducted as an analytical narrative review integrating secondary epidemiological data and the published scientific literature to explore the potential underrecognition of typhus group rickettsioses in Central America within the context of the ongoing dengue epidemic. The objective was to examine the discrepancy between clinically diagnosed dengue cases and laboratory-confirmed infections and to assess whether alternative etiologies—particularly rickettsial infections—may account for a proportion of undifferentiated febrile illnesses reported as dengue-like syndromes in the region.

Epidemiological data on dengue were obtained from the Pan American Health Organization (PAHO) Arbovirus Surveillance Portal, which compiles official national surveillance reports submitted by ministries of health across the Americas [2]. Country-level data were extracted for Central American countries (Belize, Costa Rica, El Salvador, Guatemala, Honduras, Nicaragua, and Panama) for the year 2024. Variables reviewed included total reported dengue cases, laboratory-confirmed cases, severe dengue cases, and reported deaths. These data were used to estimate the proportion of laboratory-confirmed infections relative to total reported cases and to identify potential diagnostic gaps within regional surveillance systems.

A focused review of the peer-reviewed literature was conducted to identify studies addressing the epidemiology, clinical manifestations, and diagnostic challenges associated with murine typhus and other rickettsial infections in the Americas. Publications indexed in major biomedical databases, including PubMed and MEDLINE, were reviewed, with emphasis on studies published over the past two decades. The following keywords were utilized for the literature search: ‘murine typhus’, ‘typhus group rickettsioses’, ‘*Rickettsia typhi*’, ‘rickettsial infections’, ‘dengue’, ‘arboviral diseases’, ‘acute febrile illness’, ‘Central America’, ‘South Texas’, ‘vector-borne diseases’, ‘zoonoses’, ‘flea-borne transmission’, and ‘One Health’. Eligible publications included original research articles, epidemiological reports, case series, surveillance studies, narrative and systematic reviews, as well as relevant public health reports addressing murine typhus, typhus group rickettsioses, dengue-like febrile illness, vector ecology, zoonotic reservoirs, and diagnostic challenges in the Americas. Preference was given to peer-reviewed articles with epidemiological, clinical, ecological, or public health relevance to Central America and South Texas. Articles published in English and Spanish were considered eligible for inclusion. Editorials, opinion pieces without supporting data, duplicate publications, and studies lacking relevance to the regional epidemiological context were excluded.

To contextualize these findings, a qualitative ecological comparison was performed between the epidemiological setting described in South Texas and environmental and socioeconomic conditions present across Central America. Variables considered included climatic patterns, urbanization dynamics, vector and reservoir ecology, and socioeconomic determinants such as housing conditions, waste management practices, and the presence of domestic animals in peri-domestic environments. These elements were examined using published epidemiological and ecological reports to assess the plausibility of similar transmission dynamics for *Rickettsia typhi* in Central American settings.

The analytical approach focused on identifying inconsistencies between clinically diagnosed dengue cases and laboratory-confirmed infections reported through national surveillance systems. The proportion of laboratory-confirmed dengue cases was calculated relative to total reported cases and interpreted alongside evidence describing the clinical similarity between dengue and rickettsial infections, ecological factors supporting rickettsial transmission, and structural limitations affecting diagnostic capacity in regional health systems.

This study relied exclusively on publicly available aggregated epidemiological data and the previously published scientific literature. No individual patient information or identifiable data were used; therefore, institutional ethical approval was not required.

The analysis relied on aggregated surveillance data that may be affected by underreporting, reporting delays, or variability in diagnostic practices across countries. The proportion of laboratory-confirmed dengue cases may reflect differences in diagnostic capacity rather than the true prevalence of dengue infection. Additionally, the ecological and epidemiological comparisons presented are inferential and do not constitute direct evidence of rickettsial transmission in the populations examined. Finally, the absence of systematic rickettsial testing within regional febrile illness surveillance systems limits the ability to estimate the true burden of typhus group rickettsioses in Central America. These limitations highlight the need for expanded diagnostic capacity, prospective surveillance, and seroepidemiologic studies to better characterize the contribution of rickettsial infections to dengue-like febrile illness in the region.

## 3. Results

Dengue continues to represent the foremost vector-borne disease challenge in Central America. In 2024, all four-dengue virus (DENV) serotypes were documented in circulation across the region (Table 1). A total of 543,006 suspected cases were reported, yet only 62,285 (11%) were laboratory-confirmed [2]. Current national regulations stipulate that every severe or hospitalized dengue case must have at least one blood sample collected for diagnostic confirmation. However, according to personal communications from infectious disease specialists in the region, laboratory positivity among severe cases is estimated to be approximately 50%. These findings underscore persistent gaps in diagnostic capacity and surveillance quality across Central America—gaps that call for innovative, out-of-the-box approaches to strengthen regional preparedness and response.

The published literature identified evidence supporting the circulation of rickettsial infections in Central America and neighboring regions. Confirmed severe *Rickettsia typhi* infections have been reported in Costa Rica, while regional reviews and seroepidemiological studies have documented exposure to typhus group and spotted fever group rickettsiae in several Central American countries. These findings indicate that rickettsial transmission is biologically plausible and likely underrecognized in areas where routine diagnostic testing is limited.

The reviewed literature also showed that the ecological and socioeconomic conditions favoring murine typhus transmission are present in Central America. These include abundant flea vectors, peridomestic rodents and opossums, domestic animals, informal housing, inadequate solid waste management, and warm, humid climatic conditions that support vector survival. These determinants resemble those described in endemic areas of South Texas, where murine typhus has been consistently documented.

The South Texas experience demonstrates how consistent data collection and laboratory confirmation can elucidate disease burden and transmission dynamics. Over an 18-year period, 213 pediatric cases were confirmed, showing stable annual incidence and distinct seasonal peaks [1]. Comparable systematic surveillance is lacking in Central America, where national disease control programs predominantly focus on arboviruses and malaria, leaving rickettsioses largely unmonitored. Establishing sentinel surveillance sites, incorporating rickettsial testing into febrile illness algorithms, and expanding physician training are critical steps forward. Regional reference laboratories, under the coordination of the Pan American Health Organization (PAHO), could play a pivotal role in standardizing diagnostic procedures and building workforce capacity.

Findings from South Texas also highlight the benefits of early doxycycline administration and heightened clinician awareness. In that cohort, prompt initiation of therapy correlated with shorter hospital stays and fewer complications [1]. In contrast, empirical doxycycline use for undifferentiated febrile illness in resource-limited Central American settings is often constrained by diagnostic uncertainty and antimicrobial stewardship concerns [11]. Developing clinical algorithms that integrate epidemiologic risk factors and local vector data could help reconcile these tensions, ensuring timely treatment while preserving rational antibiotic use. Pediatricians, in particular, should maintain a high index of suspicion for rickettsial disease during warm and rainy months when vector activity peaks.

## 4. Discussion

Comparable ecological and social conditions are widespread across the isthmus, where peridomestic rodents (*Rattus rattus*, *R. norvegicus*) and opossums (*Didelphis marsupialis*) serve as potential reservoirs, and *Ctenocephalides felis* is ubiquitous among cats and dogs. Although the primary focus of this review is typhus group rickettsioses caused by *Rickettsia typhi*, other flea-borne rickettsial pathogens may also contribute to dengue-like febrile illness in the region. In particular, *Rickettsia felis* infection, a member of the transitional/spotted fever group of rickettsiae transmitted by the cat flea (*C. felis*), has emerged as an underrecognized cause of acute febrile illness with clinical overlap with dengue and murine typhus [12]. The potential for zoonotic spillover is therefore considerable, especially in peri-urban environments. Moreover, climatic disruptions—such as El Niño–Southern Oscillation (ENSO) events—modulate rodent and flea populations and have been linked to increased rickettsial transmission in other endemic areas. With climate variability intensifying across Central America, these ecological interactions are expected to become more pronounced in the coming decades.

The region’s vulnerability to rickettsial diseases must also be interpreted in light of persistent health system limitations. Chronic underinvestment, weak laboratory capacity, and fragmented surveillance systems hinder early detection of emerging zoonoses. The coexistence of multiple reservoirs—rodents, opossums, and domestic animals—within densely populated human settlements further increases the probability of transmission. Poverty-driven environmental degradation and unplanned urbanization aggravate these vulnerabilities, reinforcing the need for a coordinated “One Health” approach that integrates human, veterinary, and environmental surveillance.

Despite the likely endemicity of murine typhus and related rickettsioses, most Central American countries lack reliable laboratory diagnostic capacity. National reference laboratories often rely on external confirmation through indirect immunofluorescence assays (IFA), which are costly and unavailable outside major cities. Consequently, substantial underreporting and clinical misclassification occur, as febrile illnesses consistent with rickettsial infection are frequently attributed to more common etiologies such as dengue, leptospirosis, or chikungunya. This diagnostic gap perpetuates a cycle of invisibility and delayed treatment, resulting in preventable morbidity, particularly among children.

*R. typhi* infection in adults can produce a wider and more severe spectrum of complications than historically appreciated. In addition to the classic febrile illness, adults may display significant organ involvement including acute kidney injury, hepatitis, pneumonitis, and acute respiratory distress syndrome (ARDS) when infection progresses unrecognized [7,13,14]. Central nervous system involvement is especially important, with documented meningitis, meningoencephalitis, cranial neuropathies, and prolonged post-infectious headaches [7,15,16]. Although most clinical and laboratory features fail to distinguish between typhus and viral pathogens, certain findings can point an attentive clinician toward the correct diagnosis. Hypoglycorrhachia, reported in up to 38% of typhus group rickettsial CNS infections, in the context of mild-to-moderate hyperproteinorrhachia and pleocytosis, when paired with an otherwise unremarkable neuroimaging test, remains one of the most informative early laboratory clues, helping distinguish these cases from more common viral etiologies [13,15,17]. Delayed diagnosis is strongly associated with worsened outcomes, as untreated or late-treated adult cases demonstrate higher rates of neurological sequelae and significantly increased mortality, rising from ~0.4% in uncomplicated infections to as high as 27% when CNS disease is present [7,13]. These findings reiterate the importance of early clinical suspicion and rapid initiation of doxycycline to prevent progression to severe multisystem involvement.

Madril et al., in a concise but consequential 2025 communication [18], raised an alarm by documenting seven adult cases of typhus group rickettsia CNS infection, including four confirmed and three probable. This represents the largest single-region cohort yet described in the English-language literature. One of the most striking aspects of the report is the authors’ candor that these cases were only recognized through time, accumulated experience, and a deliberate shift toward routinely including typhus in the daily differential diagnosis. The message is unmistakable: there is a learning curve for every clinician, but the first step is abandoning the deeply ingrained perception that typhus is an exotic rarity. Recognition increases case discovery, and case discovery sharpens clinical acumen, eventually reaching a tipping point where the disease is not an afterthought but a permanent fixture in diagnostic reasoning. Madril et al. [18], argue that they have reached “the end of the beginning”, the threshold at which a once “rare” disease reveals itself to be surprisingly common. Their observation is reinforced by statewide data showing typhus now accounts for 57% of all vector-borne infections in Texas [15].

The group also underscores how difficult true case capture remains [18]. Without point-of-care testing, given consent barriers, family contact challenges, and layers of bureaucratic obstruction, the seven reported cases may represent only a fraction of the true burden. Yet, the authors do maintain some amount of cautious optimism. They argue that this moment should mark the end of dismissing typhus as rare, the end of its absence from medical school curricula, and the end of its status as an overlooked, “orphaned” pathogen, with renewed urgency to develop reliable diagnostics by both public and private sectors. Only with that mindset can the pattern observed in Texas be recognized across Central America. When that transition occurs, the large proportion of cases currently attributed to dengue without confirmatory testing may finally shrink, and the longstanding diagnostic gap may reveal its true name: Typhus Group Rickettsia.

Future priorities for research and policy include the following: (1) molecular characterization of *R. typhi* and other rickettsiae in vectors and reservoirs; (2) seroepidemiologic studies among high-risk populations; (3) incorporation of rickettsial diagnostics into national febrile surveillance frameworks; and (4) evaluation of climate–vector–host interactions. Collaborative partnerships among universities, ministries of health, and international entities—such as the U.S. CDC Rickettsial Zoonoses Branch and PAHO’s regional zoonoses program—will be essential for advancing technical capacity and regional data harmonization. Concurrently, community-level interventions to reduce flea infestations in domestic animals and improve environmental sanitation can help interrupt transmission cycles.

Central America’s experiences with other reemerging infections—dengue, chikungunya, Zika, and leptospirosis—demonstrate that reactive surveillance is insufficient to protect vulnerable populations. Rickettsial diseases demand a proactive, multisectoral strategy grounded in cross-border collaboration, diagnostic readiness, and ecological understanding. The reemergence of murine typhus in Texas and its recent detection in Costa Rica are not isolated events but rather sentinel indicators of a broader regional susceptibility shaped by environmental and socioeconomic convergence. Integrating rickettsial surveillance into the regional agenda for emerging infections should therefore be considered a public health priority.

In summary, the parallels between South Texas and Central America extend beyond shared climatic and ecological features to encompass structural determinants of health vulnerability. Without enhanced diagnostic and surveillance capacity, rickettsioses will remain underrecognized, perpetuating avoidable morbidity. The work by Howard and Fergie [1] exemplifies how systematic pediatric surveillance can uncover hidden endemicity and guide targeted interventions. Adapting such frameworks for Central America represents a critical step toward regional and global health security.

## 5. Limitations

This review has several limitations. First, the analysis relied primarily on aggregated surveillance data obtained from the PAHO Arbovirus Surveillance Portal, which may be affected by underreporting, reporting delays, heterogeneity in national surveillance systems, and variability in laboratory diagnostic capacity across countries. Second, because publicly available surveillance data do not provide patient-level information, direct comparisons between laboratory-confirmed and clinically suspected dengue cases regarding severity, mortality, or clinical outcomes could not be performed. Third, the ecological and epidemiological comparisons presented between South Texas and Central America are inferential and do not constitute direct evidence of active rickettsial transmission within the populations analyzed. Fourth, the review was narrative rather than systematic in design, which may introduce selection bias in the inclusion and interpretation of the published literature. Finally, the absence of routine rickettsial testing and limited access to confirmatory diagnostics in most Central American countries significantly restrict the ability to estimate the true burden of typhus group rickettsioses in the region.

## 6. Conclusions

The low proportion of laboratory-confirmed dengue cases reported during the current Central American epidemic suggests an important diagnostic gap within regional febrile illness surveillance systems. While dengue remains a major public health threat, typhus group rickettsioses represent a plausible and underrecognized contributor to dengue-like illness in the region given the favorable ecological, climatic, and socioeconomic conditions for transmission. Limited laboratory capacity and the clinical overlap between dengue and rickettsial infections likely contribute to underdiagnosis and misclassification. Strengthening surveillance systems, expanding access to rickettsial diagnostics, and improving clinician awareness are essential to better characterize the true burden of febrile diseases in Central America and improve early recognition and treatment of neglected zoonotic infections.

## Figures and Tables

**Table 1:** Dengue cases in Central America during 2024 [2].

Countries	Dengue Cases			Proportion of Severe Dengue *	Deaths	Dengue Case Fatality Rate **
	Reported	Diagnosed by Laboratory	Severe Dengue			
Costa Rica	31,259	1301	-	-	7	22.4
El Salvador	8552	764	29	339.1	9	105.2
Belize	1148	791	4	348.4	0	-
Guatemala	188,585	6009	254	134.7	188	99.7
Honduras	177,209	10,008	2250	1269.7	160	90.3
Nicaragua	99,022	11,335	16	16.2	1	1.0
Panamá	37,231	32,077	330	886.4	52	139.7
Total	543,006	62,285	2883	530.9	417	76.8

Most dengue cases are clinically diagnosed; laboratory confirmation is low across the region (11%). * Proportion of severe dengue cases among all dengue cases × 100,000. ** Proportion of dengue deaths among all dengue cases × 100,000.

## Data Availability

No new data were created.
